# The Work of the Deptford Fund

**Published:** 1904-07-02

**Authors:** 


### THE WORK OF THE DEPTFORD FUND.

The Deptford Fund, which was founded ten years ago under the presidency of H.R.H. the Duchess of Albany, is appealing for £8,000, in order that its operations may be extended. The Fund exists for the purpose of assisting and relieving the distressed poor of Deptford, wherever possible, in conjunction with the local clergy and other workers, discretion and discrimination being exercised to avoid imposition and overlapping. The many activities include a sick-kitchen, from which last year more than 22,000 hot nourishing meat meals were distributed at a nominal payment, to sick or convalescent poor and to children, 7,320 of these being supplied at the request of the London Schools Dinner Association for necessitous children; a girls' club, with an average attendance of 255; a refuge, worked in conjunction with the Refuge and Reformatory Union; a children's guild; children's happy evenings; a school of domestic economy, with a grant from the Technical Education Board of the L.C.C., besides miscellaneous work of the nature of classes, mothers' meetings, and clothing clubs, while 185 hospital and other letters were distributed during 1903. The girls' club, beginning with a membership of 35, has outgrown its accommodation, with 360 on the roll, and a waiting list of applicants eager to join. Testimony to the value of this branch of the work was amply forthcoming at a recent meeting to promote the interests of the Fund, held at Grosvenor House, and the foreman of a large local works states that the good the club is doing is indescribable, and that it is easy to see which of the girls attend, by their manner, dress, and quiet behaviour. The domestic economy school was opened with 30 scholars in 1899, under two qualified teachers. Its purpose is to train girls from 14 to 16 years of age in cookery, laundry, needlework, dressmaking, and simple hygiene. The promoters claim that the laundry is as good as any in London, and they add that the difficulty hitherto experienced of inducing the pupils to go out to service is slowly but surely being overcome. The foundation-stone of the Albany Institute was laid by the King in 1898, and his Majesty has testified his approval of the Fund by heading the extension appeal with a donation of £25. Upwards of £800 was given or promised at Grosvenor House, when the Bishop of London made an earnest appeal to the rich to help the poor with money and personal service. The secretary is Mrs. Lamert, 24 Buckingham Palace Road, S.W.

